# mTORC2 activation protects retinal ganglion cells via Akt signaling after autophagy induction in traumatic optic nerve injury

**DOI:** 10.1038/s12276-019-0298-z

**Published:** 2019-08-13

**Authors:** Yao-Tseng Wen, Jia-Rong Zhang, Kishan Kapupara, Rong-Kung Tsai

**Affiliations:** 1 Institute of Eye Research, Hualien Tzu Chi Hospital, Buddhist Tzu Chi Medical Foundation, Hualien, Taiwan; 2 Department of Ophthalmology, Hualien Tzu Chi Hospital, Buddhist Tzu Chi Medical Foundation, Hualien, Taiwan; 3 Institute of Medical Sciences, Tzu Chi University, Hualien, Taiwan

**Keywords:** Autophagy, Retina, Cell death in the nervous system

## Abstract

Traumatic optic neuropathy is an injury to the optic nerve that leads to vision loss. Autophagy is vital for cell survival and cell death in central nervous system injury, but the role of autophagy in traumatic optic nerve injury remains uncertain. Optic nerve crush is a robust model of traumatic optic nerve injury. p62 siRNA and rapamycin are autophagy inducers and have different neuroprotective effects in the central nervous system. In this study, p62 and rapamycin induced autophagy, but only p62 siRNA treatment provided a favorable protective effect in visual function and retinal ganglion cell (RGC) survival. Moreover, the number of macrophages at the optic nerve lesion site was lower in the p62-siRNA-treated group than in the other groups. p62 siRNA induced more M2 macrophage polarization than rapamycin did. Rapamycin inhibited both mTORC1 and mTORC2 activation, whereas p62 siRNA inhibited only mTORC1 activation and maintained mTORC2 and Akt activation. Inhibition of mTORC2-induced Akt activation resulted in blood–optic nerve barrier disruption. Combined treatment with rapamycin and the mTORC2 activator SC79 improved RGC survival. Overall, our findings suggest that mTORC2 activation after autophagy induction is necessary for the neuroprotection of RGCs in traumatic optic nerve injury and may lead to new clinical applications.

## Eye disease: switching signals for repairing injured optic nerves

Regulating molecular signaling pathways that control the degradation of cellular components—a process known as autophagy—could offer a new approach to treating optic nerve damage after traumatic injuries. There is currently no established treatment option for traumatic optic nerve injury. Rong-Kung Tsai and colleagues at Tzu Chi University in Hualien, Taiwan, explored the role of a protein complex called mTORC2 in autophagy during the repair of optic nerves in rats. They investigated mTORC2 activation by small RNA molecules that also activate autophagy, and by drugs that activate autophagy but inhibit mTORC2. The results indicate that autophagy associated with activation of mTORC2 protects damaged retinal neurons and promotes visual recovery. In addition to treating optic nerve injuries, drugs activating mTORC2 and autophagy might help treat nerve-related diseases of the eye, including glaucoma.

## Introduction

Traumatic optic neuropathy (TON) is damage to the optic nerve caused directly or indirectly by assault, head trauma, disasters, or road accidents. TON results in optic nerve edema, disrupting the pial vessels and consequently limiting the vascular supply. These events trigger an inflammatory response at the injury site, causing secondary damage to the optic nerve, which leads to axon degeneration and retinal ganglion cell (RGC) death^1,2^. Currently, there are no established treatment options for TON, but optic canal decompression and corticosteroids may provide limited support in secondary optic nerve injuries^3^. Rodent optic nerve crush (ONC) is an excellent model that closely mimics human TON and exhibits similar pathophysiology, rendering it a useful tool for therapeutic and regenerative studies^4–9^.

Several studies have reported a crucial role of autophagy after optic nerve injury^10–12^. Within hours after optic nerve injury, there is a rapid increase in autophagic vesicles at the optic nerve lesion, which spread back to the RGC somata where LC3 levels are increased as early as 24 h after a lesion^11^. The transcriptional levels of autophagy-related genes such as Atg5 and Atg7 in RGC somata are increased between 3 and 10 days after a lesion^13^. The highest activation of autophagy leads to RGC death in vivo^11^. In addition, a substantial increase in p62 in the optic nerve could be caused by insufficient autophagic flux or parallel upregulation of p62^14,15^. According to our previous study, ethambutol induces impaired autophagic flux and RGC apoptosis in the rat retina^16^. In summary, autophagy is involved in RGC death after optic nerve injury. Thus, manipulation of autophagy in the injured optic nerve has therapeutic potential to preserve visual function.

mTORC1 activation results in autophagy inhibition^17^. Administering the autophagy inhibitor 3-methyladenine (3-MA) reduced axonal degeneration in the ONC model^12^. Morgan-Warren et al. demonstrated that mTOR induction could improve RGC survival after ONC^9^. By contrast, induction of autophagy by rapamycin did not protect retinal neurons in an ischemia/reperfusion model^18^. Daily intraperitoneal administration of rapamycin before optic nerve axotomy was protective for RGCs^14^. Autophagy activation has a cytoprotective role in RGCs after traumatic optic nerve injury^19^ and in TNF-induced optic nerve degeneration^20^. Thus, the actual functions and consequences of autophagy in RGCs after optic nerve injury remain controversial.

In the present study, we selected two autophagy inducers, rapamycin and p62 siRNA, to explore the role of autophagy in the ONC model. Rapamycin can inhibit mTOR expression and activate autophagy in various cells^21^. In addition, rapamycin directly inhibits mTORC1 activation; only chronic administration of rapamycin can inhibit mTORC2 but not in every cell line or tissue^22^. However, the neuroprotective effect of rapamycin remains uncertain in many optic nerve injury models. Sequestosome 1 (SQSTM1), also named P62 protein, is a ubiquitin-binding scaffold protein that colocalizes with aggregates of ubiquitinated protein; the protein is degraded by autophagic flux. P62 accumulates when autophagy is inhibited, and decreased levels can be observed when autophagy is induced^23^. p62 siRNA can facilitate autophagic flux to improve autophagy by reducing the accumulation of P62 aggregation^24^. Furthermore, p62 siRNA specifically inhibits mTORC1 assembly through a p62–raptor interaction to suppress mTORC1 activation^25^. Although p62 siRNA has not been applied clinically, several studies have revealed that p62 inhibition provides neuroprotective effects in optic nerve injury^20,24,26^. The present study investigated the role of autophagy by observing rapamycin-mediated and p62-mediated mTOR activation in a rat model of ONC.

## Methods

### Animals

Male Wistar rats (BioLASCO Co., Taiwan) weighing 150–180 g were used in this study. All animal care and surgical procedures were executed in accordance with the Association for Research in Vision and Ophthalmology Statement for the Use of Animals in Ophthalmic and Vision Research. The Institutional Animal Care and Use Committee at Tzu Chi Medical Center approved all the animal experiments. All operations were performed with the animals under anesthesia, which was achieved by intramuscular administration of a ketamine (100 mg/kg body weight) and xylazine (10 mg/kg body weight; Sigma, St. Louis, MO, USA) cocktail. The rats had free access to food and water in a controlled environment at a temperature of 23 °C and 55% humidity with a 12-h light–dark cycle.

### ONC experiments

ONC injury was induced as described in our previous reports^5–7^. Briefly, the optic nerve was exposed, and a vascular clip (60-g microvascular clip, World Precision Instruments, FL, USA) was applied at a distance of 2 mm posterior to the eyeball for 30 s. The surgery was performed carefully to avoid damage to the small vessels around the optic nerve. The rats were kept on electric heating pads at 37 °C for recovery. The sham group of rats received optic nerve exposure without the crush operation.

### Study design

For examining the neuroprotective effects of autophagy activators, ONC rats were treated with scrambled siRNA (50 pmol; *n* = 12), p62 siRNA (50 pmol; *n* = 12) or rapamycin (1 mM, Sigma-Aldrich, MO, USA; *n* = 12). To evaluate the therapeutic effect of mTORC2 activation in the rapamycin-treated group, mTORC2 activator SC79 (10 μg, Sigma-Aldrich, MO, USA; *n* = 6) was injected into rapamycin-treated rats. To investigate blood–optic nerve barrier (BOB) protection by Akt activation, PI3K/Akt inhibitor LY294002 (10 μg, EMD Millipore corp., MA, USA; *n* = 3) was administered to p62-siRNA-treated rats. All siRNAs were purchased from GeneDireX (Las Vegas City, NV, USA). As mentioned, a single shot of drugs was administered through intravitreal injection immediately after ONC procedures. Twelve rats without ONC operation were allocated to the sham group. Rats were euthanized at the second week post-ONC for further analysis.

### Immunostaining for autophagy markers in the retina

Retinal sections were first stained with LC3 and LAMP1 antibodies. The primary antibodies LC3 (1:100; Santa Cruz Biotechnology, Inc., USA) and LAMP1 (1:100, Abcam, Cambridge, MA, USA) were used. The secondary antibodies FITC (1:100; Kirkegaard and Perry, MD, USA) and Alexa 568 (1:200; Invitrogen) were used. Cell nuclei were counterstained with 4’,6-diamidino-2-phenylindole (DAPI; 1:1000; Sigma, St. Louis, MO, USA).

### Quantitative reverse transcription-polymerase chain reaction

Arg 1, CD206, and Fizz1 are markers of M2 macrophages^27^. The Qiagen RNeasy Mini Kit was used to isolate tissue RNA from optic nerve lysates obtained through the sonication method. All RNA samples were reverse transcribed for 30 min at 42 °C with a High-Capacity cDNA Reverse Transcription Kit according to the standard protocol of the supplier (Applied Biosystems, Foster City, CA, USA). Quantitative reverse transcription-polymerase chain reaction (qRT-PCR) was performed using an AB PRISM 7300 Sequence Detection System (Applied Biosystems, CA, USA) with the QuantiTect SYBR green qRT-PCR kit (Qiagen, Hilden, Germany). The expression levels of each gene were normalized to the reference gene, CypA. The forward and reverse primers used in this study are provided in the supplementary file (Table S1).

### Flash visually evoked potentials (FVEPs)

Flash visual evoked potentials (FVEPs) were recorded 2 weeks after ONC. A visual electrodiagnostic system (Espion, Diagnosys LLC, Gaithersburg, MA, USA) was used to measure FVEPs. The first positive wavelet was defined as the P1 wave, and the first negative wavelet was defined as the N1. The amplitudes of the P1-N2 wave were compared among the groups (*n* = 6 rats in each group) to estimate visual function^28^.

### Retrograde labeling of RGCs with Fluoro-Gold

The retrograde labeling of the RGCs was performed 1 week before the rats were euthanized. The detailed procedure has been described in our previous reports^4–7,28^. Briefly, 2 μL of 5% Fluoro-Gold (Fluorochrome LLC, CO, USA) was injected into each superior colliculus. After euthanasia, RGCs were measured at a distance of 1 or 3 mm from the center of the optic nerve head to provide the central and midperipheral RGC densities, respectively. We calculated the numbers of RGCs in five randomly selected areas measuring 62,500 μm^2^ each in the central and midperipheral regions of each retina, and we estimated their averages as the mean densities of RGCs in the central and midperipheral regions of each retina (*n* = 6 in each group).

### In situ nick end labeling assay for apoptotic cell measurements

Frozen sections were prepared with retinal samples cut at 1–2 mm sagittal from the optic nerve head to ensure the use of similar fields for comparison.^29^ An in situ nick end labeling (TUNEL) assay (DeadEndFluorometric TUNEL System, Promega Corporation, Madison, WI, USA) was used to detect apoptotic cells. TUNEL-positive cells in the RGC layer of each sample were counted in 10 high-powered fields (HPFs, ×400 magnification).

### Immunostaining at the injury site of optic nerves

The ED1 antibody reacted against extrinsic macrophages and intrinsic microglia^28,30,31^. Monoclonal antibodies against ED1 (1:100, Abcam, Cambridge, MA, USA) were used. Arg1 and CD206 are M2 macrophage markers^32^. Polyclonal antibodies against Arg1 and CD206 (1:100, Abcam, Cambridge, MA, USA) were used to determine macrophage polarization. The samples were incubated in a primary antibody overnight at 4 °C. The secondary antibody conjugated with fluorescein isothiocyanate (FITC and rhodamine, 1:100, Jackson ImmunoResearch Laboratories, West Grove, PA, USA) was incubated at room temperature for 1 h. Counterstaining was performed using DAPI (1:1000, Sigma, St. Louis, MO, USA). For comparison, the ED1-positive cells were calculated in six HPFs (×400 magnification) at the optic nerve lesion site.

### Western blotting

Retinal samples were collected, homogenized, and then centrifuged at 15,000 × *g* for 15 min at 4 °C. Western blotting was used to measure the levels of LC3 (Santa Cruz Biotechnology, Inc., USA), LAMP1 (Abcam, Cambridge, MA, USA), p-mTOR S2448 (Cell Signaling, Beverly, MA, USA), p-S6K (Cell Signaling, Beverly, MA, USA), p-Rictor (Cell Signaling, Beverly, MA, USA), p-Akt S473 (Cell Signaling, Beverly, MA, USA), and p-GSK3b S9 (Cell Signaling, Beverly, MA, USA) in retinal samples. Retinal protein extracts were separated using a 4–12% NuPAGE Bis-Tris gel (Invitrogen, Carlsbad, CA, USA). The separated proteins were transferred onto polyvinylidene difluoride membranes and blocked with 5% milk in Tris-buffered saline/Tween-20 containing 20 mM Tris-HCl (pH 7.5), 0.5 M NaCl, and 0.5% Tween-20. The membranes were then blotted with mouse anti-LC antibodies, mouse anti-LAMP1 antibody, mouse anti-p62 antibody, mouse anti-mTOR antibody, and goat anti-mouse immunoglobulin (Abcam, Cambridge, MA, USA). The blots were then developed with Enhanced Chemiluminescent Substrates (Perkin-Elmer Life Science, Boston, MA, USA), and the relative intensities of the bands were measured using an image analysis system (Amersham Biosciences Uppsala, Sweden).

### Transmission electron microscopy

The ultrastructure of the capillary tight junction was observed as described in our previous report^29^. Sections measuring 80 nm in thickness were observed under a transmission electron microscope (Hitachi High-Technologies Corporation, Japan). Five to six images were taken per sample at the desired magnification.

### Statistical analysis

All measurements in this study were performed in a masked fashion. The Mann–Whitney *U* test was used to evaluate the differences among groups in all experiments. Data are reported as the mean ± standard deviation (SD), and statistical significance was confirmed if *p* < 0.05. All statistical analyses were performed using commercial software (SPSS, Chicago, IL, USA).

## Results

### p62 knockdown and rapamycin induce autophagy in the retina after ONC

**Fig. 1:**
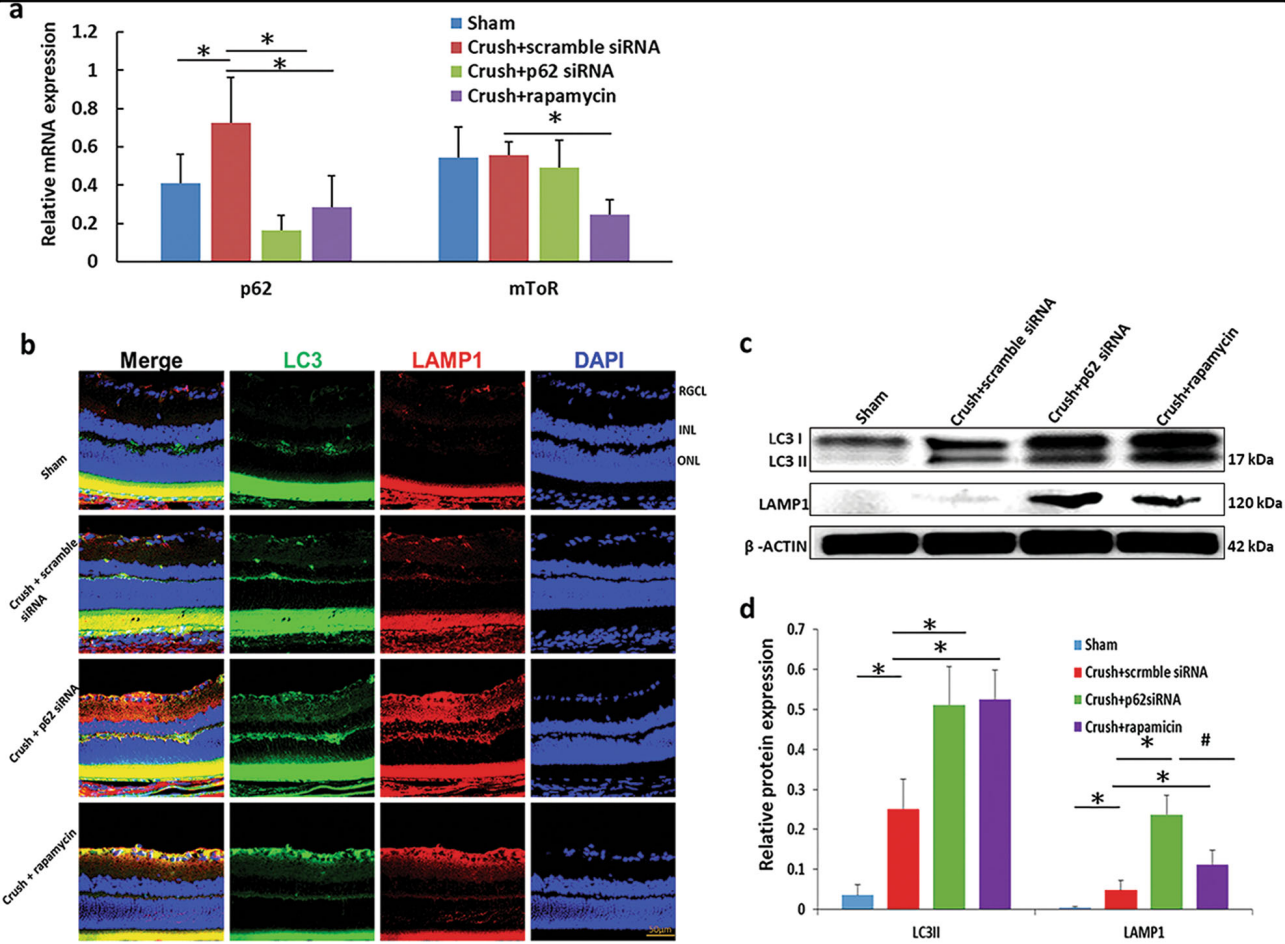
Confirmation of autophagy induction after treatment with p62 siRNA or rapamycin. **a** mRNA levels of p62 and mTOR in the retina tissue for each group (*n* = 6). Both autophagy inducers reduced the mRNA level of p62 but only rapamycin reduced mRNA mTOR expression. **b** Immunostaining for LC3 and LAMP1 expression in the retinal section to evaluate autophagy induction. Treatment with either p62 siRNA or rapamycin induced a higher level of LC3 and LAMP1 than did treatment with scrambled siRNA. **c** Protein expression levels of LC3 II and LAMP1 in the retina. Representative results from three independent experiments are shown. **d** Quantitative analysis of the level of LC3 II and LAMP1 normalized to the internal control, beta-actin. The levels of LC3-II and LAMP1 in retinas were increased in the p62-siRNA-treated group and the rapamycin-treated group compared with those in the scrambled siRNA-treated group. Treatment with p62 siRNA induced more LAMP1 expression than did treatment with rapamycin. *P < 0.05 indicates scrambled siRNA-treated group versus other groups; ^#^*P* < 0.05 indicates the P62--siRNA-treated group versus rapamycin-treated group. RGCL retinal ganglion cell layer, INL inner nuclear letter, ONL outer nuclear layer

The autophagy induction efficacy of p62 siRNA and rapamycin was determined according to the mRNA levels of p62 and mTOR in retinas at 1 week after ONC. Both autophagy inducers reduced the mRNA expression level of p62 in the retina (Fig. 1a). Treatment with rapamycin reduced mTOR mRNA expression. However, this result was not observed in the p62-siRNA-treated group (Fig. 1a). In autophagy activation, three main steps must be triggered: the formation of an autophagosome; fusion of the autophagosome and a lysosome, also called an autolysosome; and degradation of the autolysosome. The autophagy marker protein LC3 is a readout for triggering the formation of an autophagosome. The autophagy marker protein LAMP1 is considered to be an indicator of the formation of an autolysosome. These two autophagy markers must be inspected when monitoring the condition of autophagy flux. Compared with treated with scrambled siRNA, treatment with either p62 siRNA or rapamycin induced higher levels of LC3 and LAMP1 (Fig. 1b). However, compared with treatment with scrambled siRNA, treatment with rapamycin only induced LC3 and LAMP1 expression in the RGC layer. Treatment with p62 siRNA induced LC3 and LAMP1 expression from the RGC layer to the outer nuclear layer in retinas. The expression of LC3 and LAMP1 demonstrated that autophagy was activated throughout the retina with p62 siRNA treatment compared with that observed after rapamycin treatment in ONC. To validate our findings, we performed Western blotting for LC-II and LAMP1. The levels of LC3-II in the retinas were increased by approximately twofold in the p62-siRNA-treated group and rapamycin-treated group compared with those in the scrambled siRNA-treated group (*p* < 0.05) (Fig. 1c). Treatment with p62 siRNA and rapamycin increased the LAMP1 expression level in retinas by approximately 5-fold and 2.5-fold, respectively (*p* < 0.05) (Fig. 1d). In addition, compared with treatment with rapamycin, treatment with p62 siRNA increased the LAMP1 expression level by approximately twofold (*p* < 0.05).

### Evaluation of visual function by FVEPs

**Fig. 2:**
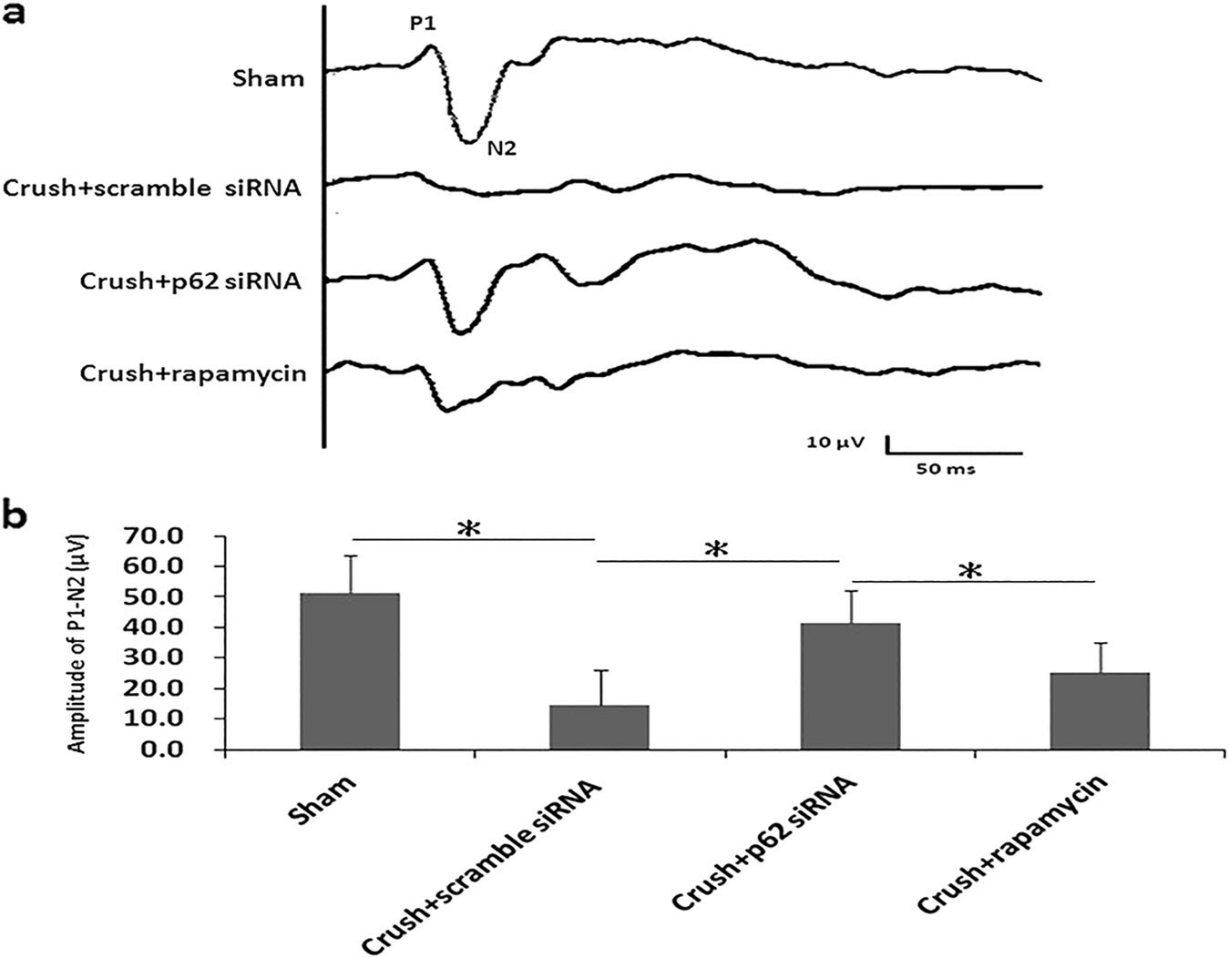
Visual function preservation after treatment with p62 siRNA and rapamycin 2 weeks after ONC. **a** Representative FVEP tracings 2 weeks after ONC in the sham group, scrambled siRNA-treated group, p62 siRNA-treated group, and rapamycin-treated group. **b** Bar charts showing the P1-N2 amplitude. Data are expressed as the mean ± SD in each group (*n* = 6). The amplitudes of the P1-N2 waves in the p62 siRNA-treated group were significantly higher than those in the scrambled siRNA-treated group and rapamycin-treated group. *P < 0.05

The FVEPs of the sham group and scrambled siRNA-treated, p62-siRNA-treated, and rapamycin-treated groups were recorded (Fig. 2a). The latency of the P1 wave was not significantly different among the groups in the FVEP tests. The amplitudes of the P1-N2 waves in the scrambled siRNA-treated, p62-siRNA-treated, and rapamycin-treated groups were 14.6 ± 11.6, 41.5 ± 10.5, and 25.2 ± 9.8 μV, respectively. The P1-N2 amplitudes in the p62-siRNA-treated group were significantly higher than those in the scrambled-siRNA-treated group and rapamycin-treated group. However, the amplitudes of the P1-N2 waves showed no significant difference between the rapamycin-treated group and scrambled-siRNA-treated group (Fig. 2b).

### Only p62 siRNA treatment preserves the RGC survival rate and rescues RGCs from apoptosis

**Fig. 3:**
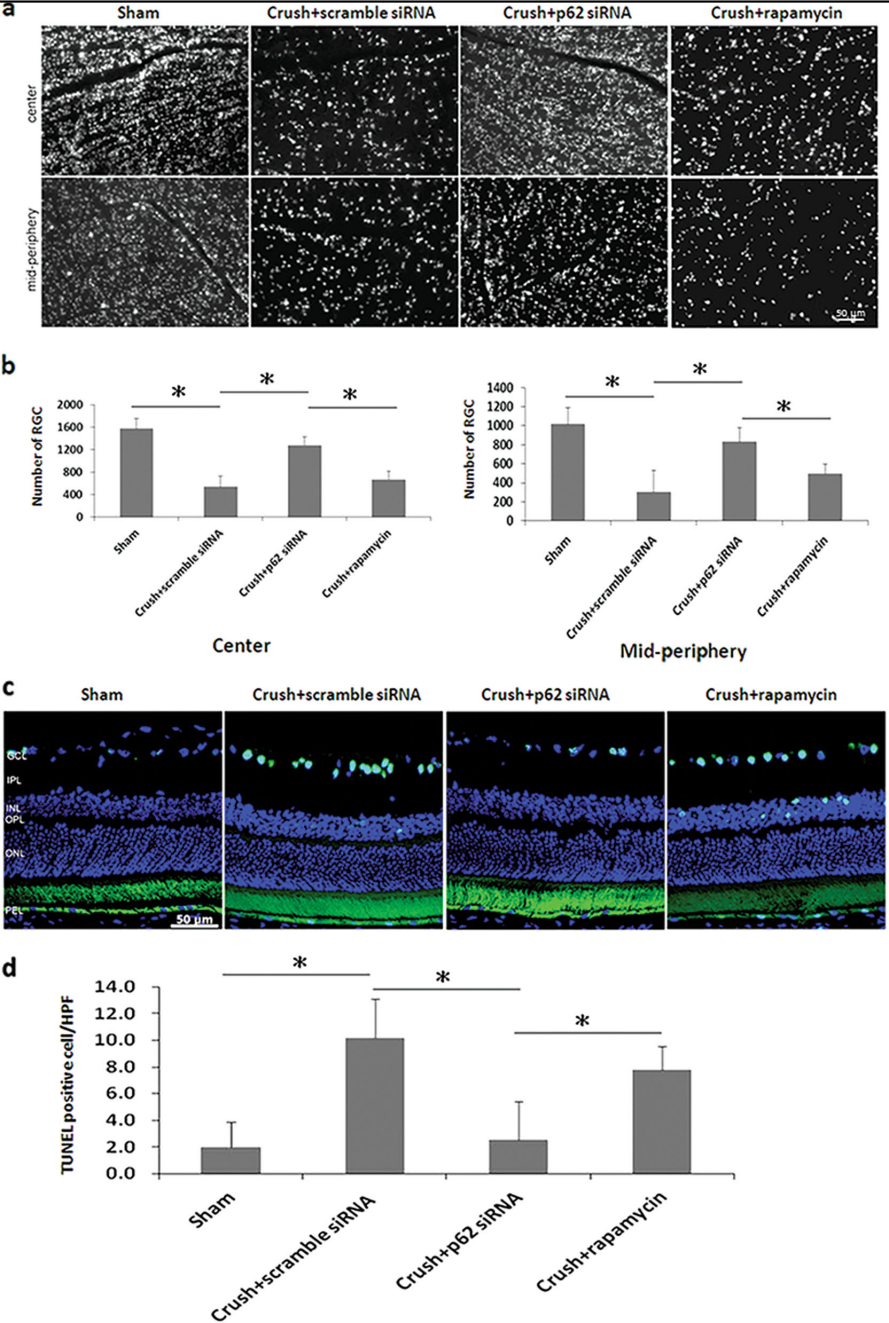
p62 siRNA treatment preserved a higher number of RGCs than rapamycin treatment did 2 weeks after ONC. **a** Representatives of flat-mounted central and midperipheral retina and the morphometry of RGCs labeled by retrograde tracer in each group (*n* = 6 per group). **b** RGC density of the central retina and midperipheral retina in each group. Data are expressed as the mean ± SD in each group (*n* = 6). The numbers of RGCs in the central and midperipheral retina in the p62 siRNA-treated group were 1.91-fold and 1.67-fold higher than those in the rapamycin-treated group, respectively. **c** Representative pictures of apoptotic cells in the RGC layer of each group. Stronger antiapoptotic effects were observed in the p62 siRNA-treated group than in the rapamycin-treated group 2 weeks after ONC. Apoptotic cells (TUNEL-positive cells/HPF; green) overlapped with nuclei of RGCs (blue) in the RGC layer. **d** Quantification of TUNEL-positive cells per HPF. Data are expressed as the mean ± SD in each group (*n* = 6). The apoptotic cells in the p62 siRNA-treated group were significantly reduced by approximately 3.99-fold and 3.08-fold compared with those in the scrambled siRNA-treated group and rapamycin-treated group, respectively. *P < 0.05

Treatment with p62 siRNA preserved a higher density of RGCs in the central and midperipheral retina than did treatment with scrambled siRNA (Fig. 3a). The RGC densities in the central retinas of the sham group and scrambled siRNA-, p62-siRNA-treated, and rapamycin-treated groups were 1576 ± 185, 536 ± 196, 1281 ± 148, and 673 ± 144 cells/mm^2^, respectively. The RGC densities in the midperipheral retinas of the sham group and scrambled siRNA-, p62-siRNA-, and rapamycin-treated groups were 1017 ± 173, 301 ± 230, 832 ± 148, and 496 ± 96 cells/mm^2^, respectively (Fig. 3b). The numbers of RGCs in the central and midperipheral retina in the p62-siRNA-treated group were 2.39-fold and 2.76-fold higher than those in the scrambled-siRNA-treated group, respectively. The numbers of RGCs in the central and midperipheral retinas in the p62-siRNA-treated group were 1.91-fold and 1.67-fold higher than those in the rapamycin-treated group, respectively. There was no significant difference in the numbers of RGCs between the rapamycin-treated group and the scrambled-siRNA-treated group.

The numbers of TUNEL-positive cells in the RGC layer of the sham group and scrambled siRNA-treated, p62-siRNA-treated, and rapamycin-treated groups (Fig. 3c) were 2.0 ± 1.9, 10.2 ± 2.9, 2.5 ± 2.8, and 7.8 ± 1.7, respectively (Fig. 3d). The apoptotic cells in the p62-siRNA-treated group were significantly reduced by 3.99-fold and 3.08-fold compared with those in the scrambled-siRNA-treated group and rapamycin-treated group, respectively (Fig. 3d). Similar to the RGC survival rate, there was no significant difference in apoptotic RGCs between the rapamycin-treated group and scrambled siRNA-treated group.

### Status of extrinsic macrophages and microglia after autophagy induction in the optic nerve

**Fig. 4:**
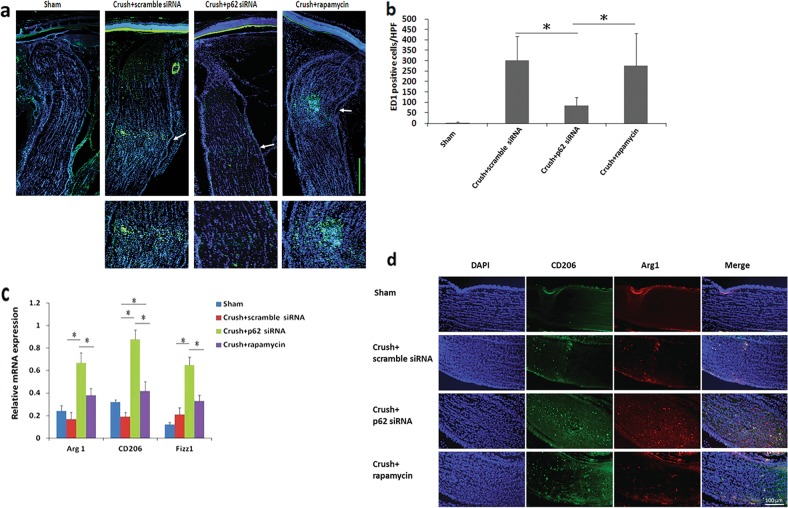
Macrophage infiltration in the p62 siRNA-treated group was lower than that in the rapamycin-treated group 2 weeks after ONC. **a** Representative figures for ED1 staining in the longitudinal sections of the optic nerve. The ED1-positive cells (macrophages) in green were labeled with FITC, and the nuclei in blue were stained with DAPI. White arrows in the top panel are the crush site, magnified images of the crush site are given below the corresponding panels (**b**) Quantification of ED1-positive cells/HPF. Data are expressed as the mean ± SD in each group (*n* = 6). The macrophage recruitment in the p62 siRNA-treated group was reduced by 3.52-fold and 3.26-fold compared with that in the scrambled siRNA-treated group and rapamycin-treated group, respectively. **c** Evaluation of M2 macrophage polarization after autophagy inducer treatment in the ONC model. Relative mRNA expression levels of the markers of M2 macrophages in the optic nerves are shown as histograms. Each value was normalized to the reference gene, CypA. The expression levels of Arg 1, CD206, and Fizz1 (markers of M2 macrophages) were increased in the optic nerve after immediate treatment with p62 siRNA compared with those observed after treatment with scrambled siRNA or rapamycin. Treatment with rapamycin also induced CD206 expression, in contrast to treatment with scrambled siRNA. **d** Immunostaining of CD206 and Arg1 in the optic nerves at 2 weeks after ONC for evaluating M2 macrophage polarization. CD206 and Arg1 were highly expressed in the optic nerve following treatment with p62 siRNA compared with those observed following treatment with scrambled siRNA or rapamycin. *P < 0.05

The levels of extrinsic macrophages in the optic nerve indicate disruption of the BOB^28,29^, in addition to the anti-inflammatory efficacy of the neuroprotective agent. Moreover, microglia polarization toward the M2 state indicates anti-inflammatory activity^28^. The numbers of ED1-positive cells in the sham group and the scrambled siRNA-treated, p62-siRNA-treated, and rapamycin-treated groups were 2.7 ± 4.6, 301.3 ± 115.4, 85.7 ± 39.6, and 277.0 ± 154.6, respectively (Fig. 4a, b). The macrophage recruitment in the p62-siRNA-treated group was reduced by 3.52-fold and 3.26-fold compared with that in the scrambled siRNA-treated group and rapamycin-treated group, respectively. Furthermore, qRT-PCR results showed that Arg 1, CD206, and Fizz1 (markers for M2 microglia) were increased in the optic nerve after treatment with p62 siRNA compared with those observed after treatment with scrambled siRNA or rapamycin (Fig. 4c). Compared with treatment with scrambled siRNA, treatment with rapamycin also induced CD206 expression. In immunostaining analysis, the levels of CD206 and Arg1 were highly expressed in the optic nerve after immediate treatment with p62 siRNA compared with those observed after treatment with either scrambled siRNA or rapamycin (Fig. 4d). These results show that p62 knockdown halts macrophage infiltration and induces M2 polarization to a greater extent than rapamycin treatment does.

### mTORC2-mediated Akt activation is necessary for RGC survival after ONC

**Fig. 5:**
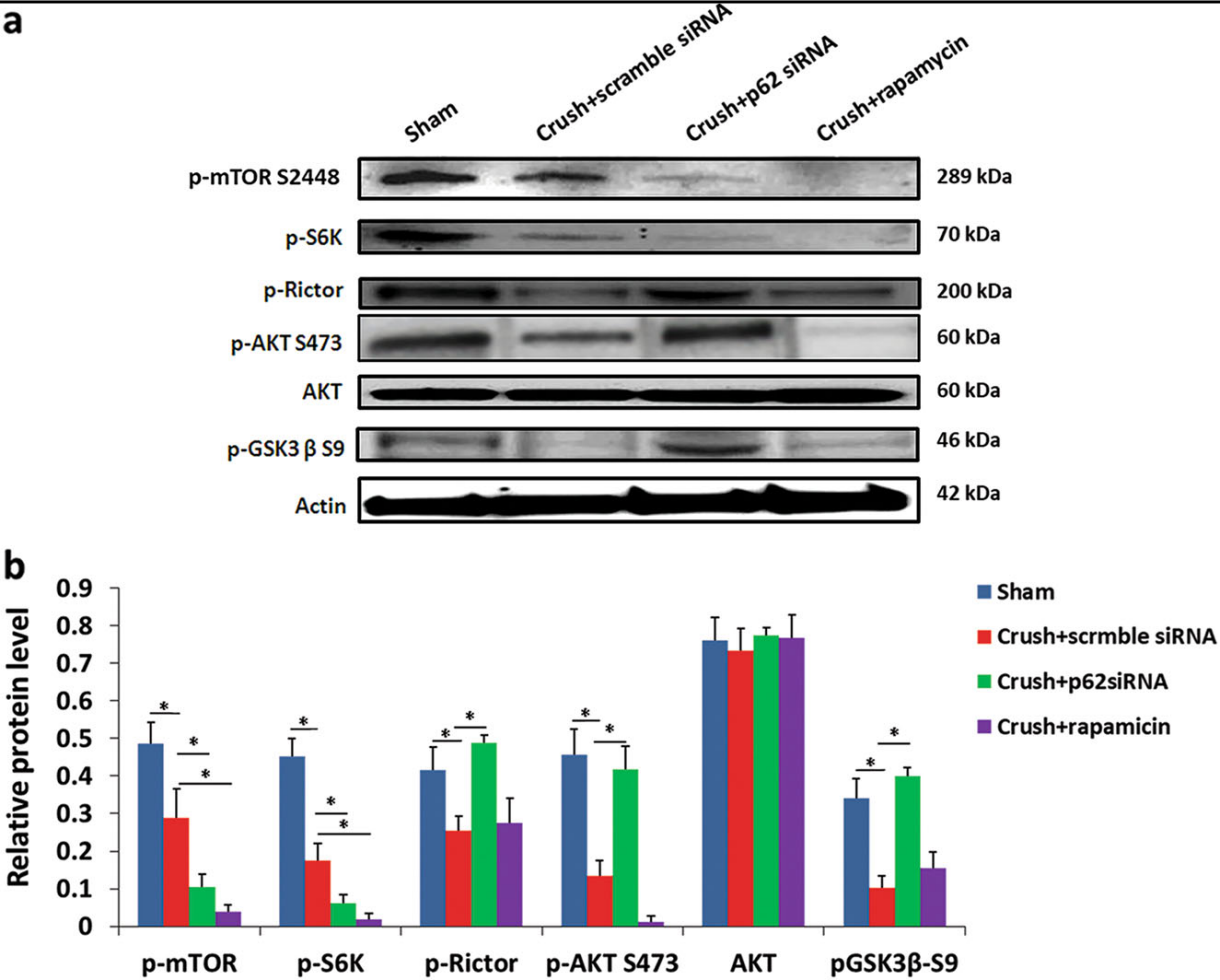
Regulation of mTOR C1 and mTOR C2 activation in the retina after autophagy inducer treatment. **a** Western blotting of the expression levels of p-mTOR, p-S6K, p-Rictor, p-Akt S473, Akt, and p-GSK3β in each group. Representative results from three independent experiments are shown. **b** Quantitative analysis of the levels of p-mTOR, p-S6K, p-Rictor, p-Akt S473, Akt, and p-GSK3β normalized to the internal control, β-actin. *P < 0.05

mTOR activation was inhibited by treatment with either p62 siRNA or rapamycin after ONC (Fig. 5a, b). The activation of S6K (downstream of mTORC1) in the p62 siRNA-treated and rapamycin-treated groups was 2.81-fold and 9.67-fold lower than that in the scrambled siRNA-treated group, respectively. The phosphorylation of Rictor, a key component of mTORC2, in the p62-siRNA-treated group was 1.91-fold and 1.77-fold higher than that in the scrambled siRNA-treated and rapamycin-treated groups, respectively. We observed no significant activation of Rictor in the rapamycin-treated group compared with that in the scrambled siRNA-treated group. The phosphorylation of Akt in the p62 siRNA-treated group was 3.14-fold and 34.83-fold higher than that in the scrambled siRNA-treated group and rapamycin-treated group, respectively. The phosphorylation of GSK3β-S9 in the p62 siRNA-treated group was 3.93-fold and 2.59-fold higher than that in the scrambled siRNA-treated group and rapamycin-treated group, respectively. These observations imply that p62 knockdown exerts neuroprotection via mTORC2-mediated Akt activation; however, rapamycin inhibited both mTORC1 and mTORC2, which resulted in reduced neuroprotective effects. Our previous findings showed BOB breakdown in the acute phase of retinal ischemia^29^. Hence, to evaluate the role of Akt activation in the acute phase of ONC, the capillary endothelial tight junction at the ONC site was examined. Compared with sham (Fig. S1a), one day after ONC, the loss of the tight junction was observed in the capillaries of optic nerves (Fig. S1b). Treatment with p62 siRNA preserved the tight junction on day one after ONC (Fig. S1c). Combined treatment with p62 siRNA and Akt inhibitor did not preserve the tight junction one day after ONC (Fig. S1d), indicating that Akt activation is a critical process along with mTORC2 activation. The rapamycin-treated group was not examined for BOB integrity because treatment with rapamycin did not prevent macrophage infiltration in the optic nerve. Furthermore, compared with treatment with rapamycin alone, combined treatment with rapamycin and mTORC2 activator SC79 preserved the RGC survival rate in the central and midperipheral retins (Fig. S2a). The numbers of RGCs in the central and midperipheral retinas in the combined treatment group were 1.72-fold and 1.70-fold higher than those in the rapamycin-treated group, respectively (Fig. S2b).

## Discussion

This study showed that intravitreal injections of an autophagy inducer (p62 siRNA) in an ONC model preserved more visual function than did treatment with another autophagy inducer (rapamycin) or scrambled siRNA. As expected, treatment with p62 siRNA rescued more RGCs and reduced more apoptotic RGCs in the retina than did treatment with rapamycin. Treatment with p62 siRNA had a more significant potency on autophagy induction than did treatment with rapamycin, as demonstrated by higher LAMP1 expression throughout the retina. Compared with rapamycin treatment, treatment with p62 siRNA significantly reduced macrophage infiltration into the optic nerve. Treatment with rapamycin reduced mTORC1 and mTORC2 activation. By contrast, p62 siRNA administration specifically inhibited mTORC1 activation and maintained mTORC2 activation after ONC. The activation of mTORC2 led to the phosphorylation of the Akt*/*GSK*-*3β signaling pathway. GSK-3β phosphorylation via the PI3K/Akt pathway can reduce neuroinflammation and stabilize the blood–brain barrier (BBB)^33–35^. Therefore, we conclude that treatment with p62 knockdown maintained mTORC2-mediated Akt activation to protect BOB integrity after ONC.

Both treatments induced M2 microglia/macrophage phenotypes, but treatment with p62 knockdown polarized more M2 microglia/macrophage than did treatment with rapamycin. Toll-like receptor 2 signaling triggers M2 polarization and leads to NF-κB p65 degradation via a lysosome-dependent pathway^36^. This NF-κB p65-containing aggresome-like structure is recognized by p62/SQSTM1 and degraded by selective autophagy^36^. Thus, autophagy induction may facilitate NF-κB p65 degradation through p62 inhibition to improve M2 macrophage polarization. In sum, p62 knockdown reduced macrophage infiltration and neuroinflammation in the optic nerve to inhibit cytokine-induced apoptosis of RGCs through dual actions: mTORC2 activation stabilizing the BOB and M2 macrophage polarization.

The p62–raptor interaction elucidates why cells need p62 to activate mTORC1 in response to cell stimulation by amino acids^25^. Thus, treatment with p62 knockdown selectively inhibits mTORC1 assembly. Our data also show that both autophagy inducers could induce autophagy and inhibit mTORC1. Although the functional association between autophagy and apoptosis remains unclear, a small connection shows that p62 levels are upregulated by autophagy deficiency or impairment^10,23,37^. Upregulation of p62 in the setting of autophagy impairment increases caspase-8-dependent apoptosis to improve its self-aggregation/activation and apoptosis^38^. Thus, treatment with p62 siRNA may inhibit p62 self-aggregation after optic nerve injury to prevent RGC apoptosis.

Research has reported that pretreatment of rapamycin by intraperitoneal injections can inhibit mTOR phosphorylation and preserve RGCs^8,39^. This slight preservation of RGCs is different from our observations. Another study on optic nerve axotomy also demonstrated that intraperitoneal injections of rapamycin preserved approximately 20% of RGCs on day 10 after optic nerve axotomy^14^. These studies have applied the same approach as intraperitoneal injections of rapamycin. Intraperitoneal injection of rapamycin before optic nerve injury induction may suppress the immune response by disrupting cytokine signaling^40^ to reduce optic nerve inflammation-induced damage. Morgan-Warren et al. reported that rapamycin reduced the RGC neuroprotective effects of siRTP801 in vitro^9^. Autophagy induction by intravitreal rapamycin administration did not provide RGC protection against ischemic stress^18^, which is consistent with our findings. This result suggests that intravitreal injection of rapamycin after optic nerve damage may not provide enough anti-inflammatory and immunosuppressive effects to protect RGCs.

Consistent with our findings, previous studies found that mTOR pathway modulation can assist axon regeneration after optic nerve injury through genetic deletion of PTEN^41^ and delivery of fibroblast-derived exosomes^42^. Our strategy is slightly different from that in previous studies because we used autophagy activators to modulate the mTOR pathway. Given the importance of the mTOR pathway, we further narrowed down the pathway involved in the activation of different mTOR complexes. Depending on the type and function of the autophagy inducer, mTOR modulation may vary to different extents. In this study, rapamycin inhibited not only mTORC1 activation but also mTORC2 activation. Inhibition of mTORC2 activation further resulted in Akt and GSK3β inactivation. Akt inactivation led to BOB disruption, which caused macrophage infiltration into the optic nerve. GSK3β phosphorylation develops as an essential target for the stabilization of the BBB in neuroinflammation^43^. mTORC2 affects biological processes through feedback phosphorylation of Akt, which in turn regulates cell survival mediators and other growth factors, such as GSK3β, and components of mitochondrial apoptosis machinery^44^. Several studies have also revealed a strong association of GSK3β phosphorylation with enhanced neuronal growth^45–48^. Consistent with our observations, only P62 knockdown, without inhibition of mTORC2-mediated AKT-GSK3β signaling, provided antiapoptotic effects in the ONC model.

**Fig. 6:**
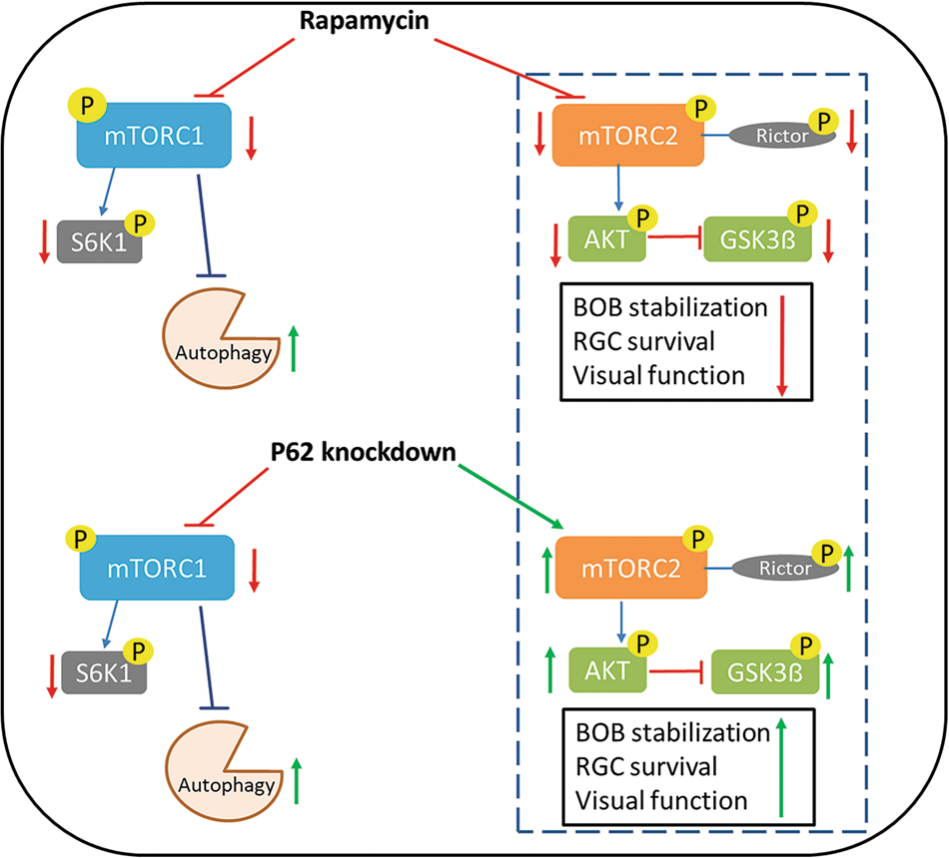
Summary of the role of autophagy inducer in the ONC model. Two autophagy inducers showed a dramatic difference in neuroprotective effects in the ONC model. Treatment with p62 siRNA results in a favorable neuroprotective effect by triggering specific mTORC1 deactivation and maintaining mTORC2 activation, which leads to the reduction in neuroinflammation through BOB protection and M2 macrophage polarization. Rapamycin can activate autophagy but provides no protection in the ONC model because the inactivation of mTORC2 results in BOB disruption, which may induce macrophage infiltration and pro-inflammatory cytokine-induced optic nerve damage

In conclusion, the neuroprotective effects caused by treatment with p62 siRNA involve specific mTORC1 inhibition and mTORC2 activation, which leads to BOB stabilization and M2 macrophage polarization. Treatment with rapamycin can activate autophagy but does not protect RGCs in the ONC model because of the inactivation of mTORC2 inhibition (Fig. 6). This finding suggests an essential role of mTORC2 activation and fills a knowledge gap in the literature on autophagy. Because autophagy is involved in other retinal diseases, such as glaucoma and optic neuropathies, autophagy regulation by using external agents may emerge as a promising clinical application.

## Supplementary information

Supplementary Materials
