# Supplementary material for: mTORC2 activation protects retinal ganglion cells via Akt signaling after autophagy induction in traumatic optic nerve injury
Source: Exp Mol Med. 2019 Aug 13;51(8):96. doi: 10.1038/s12276-019-0298-z (PMC6802655; doi:10.1038/s12276-019-0298-z)
Supplement: Supplementary file 1 — Supplementary Materials [file 12276_2019_298_MOESM1_ESM.docx]

# mTORC2 activation protects retinal ganglion cells via Akt signaling after autophagy induction in traumatic optic nerve injury

Yao-Tseng Wen^1*^, Jia-Rong Zhang^2*^, Kishan Kapupara^1^, Rong-Kung Tsai^1,3^

*^1^Institute of Eye Research, Buddhist Tzu Chi General Hospital, Hualien, Taiwan*

*^2^Department of Ophthalmology, Buddhist Tzu Chi General Hospital, Hualien, Taiwan*

*^3^Institute of Medical Sciences, Tzu Chi University, Hualien, Taiwan*

*Both authors have equal contribution to this project.

Yao-Tseng Wen, E-mail: ytw193@gmail.com

Jia-Rong Zhang, E-mail: yyrock04@gmail.com

Kishan Kapupara, E-mail: contactkishankapupara@gmail.com

**Corresponding author:**

Rong-Kung Tsai MD, Ph.D., Institute of Eye Research, Buddhist Tzu Chi General Hospital, Tzu Chi University, 707 Sec. 3 Chung-Yung Road, Hualien 970, Taiwan.

E-mail: rktsai@tzuchi.com.tw

Tel.: 886-3-8561825 ext. 2112, Fax: 886-3-8577161

The authors declare that they have no competing interests

**Table S1.** List of primers used in this study

| **Gene** | **Forward (F) and reverse primers(R)** |
| --- | --- |
| P62 | TCCCTGTCAAGCAGTATCC (F) |
|  | TCCTCCTTGGCTTTGTCTC (R) |
| mTOR | TTGAGGTTGCTATGACCAGAGAGAA |
|  | TTACCAGAAAGGACACCAGCCAATG |
| Arg1 | TCGGAGCGCCTTTCTCTAAG |
|  | ATCCCCGTGGTCTCTCACAT |
| Cd206 | AACGTTCGCTGATGCAAACC |
|  | TGTAAACTGCACCTGCTCGT |
| Fizz1 | CAACAGGATGAAGACTGCAACCT |
|  | GGGACCATCAGCTAAAGAAG |
| CypA | CACCGTGTTCTTCGACATCAC |
|  | CCAGTGCTCAGAGCACGAAAG |

**Supplementary figures**

**
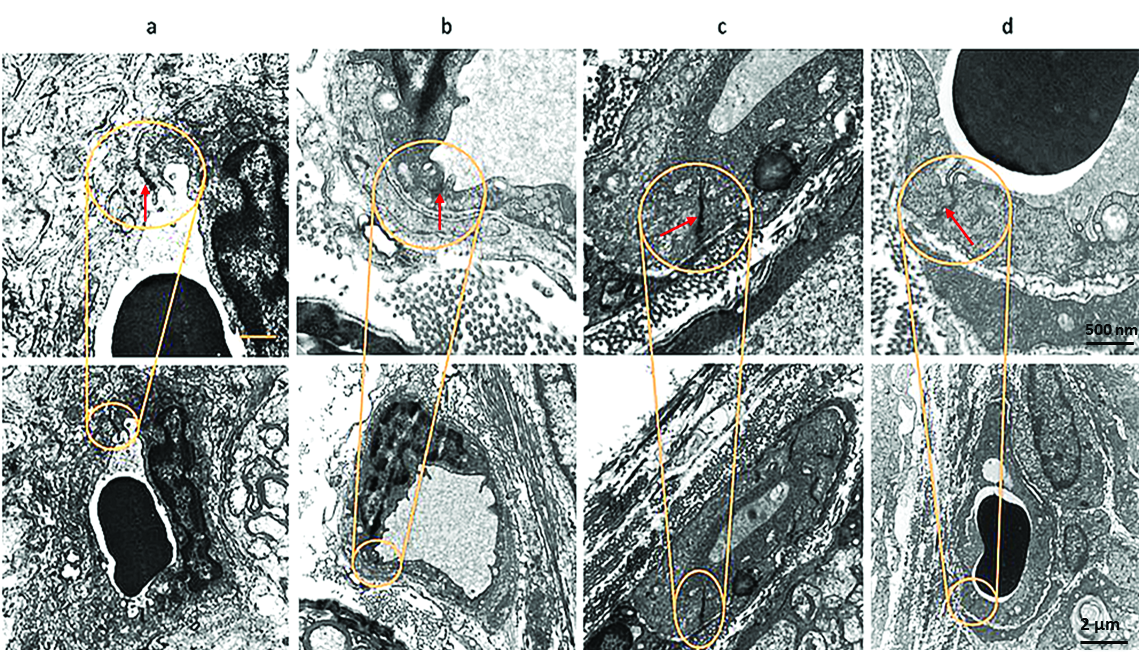
**

**Figure S1.** Preservation of BOB integrity after ONC by treatment with p62 siRNA via AKT activation. The TEM on day 1 after ONC, (**a**) capillary tight junction of the sham group, (**b**) disrupted tight junction after scramble siRNA treatment (**c**) preserved tight junction after p62 siRNA treatment, and (**d**) combined treatment with p62 siRNA and AKT inhibitor-treated group shows disruption of tight junction indicating involvement of AKT in BOB maintenance. The yellow circle with red arrows indicates the endothelial tight junction.


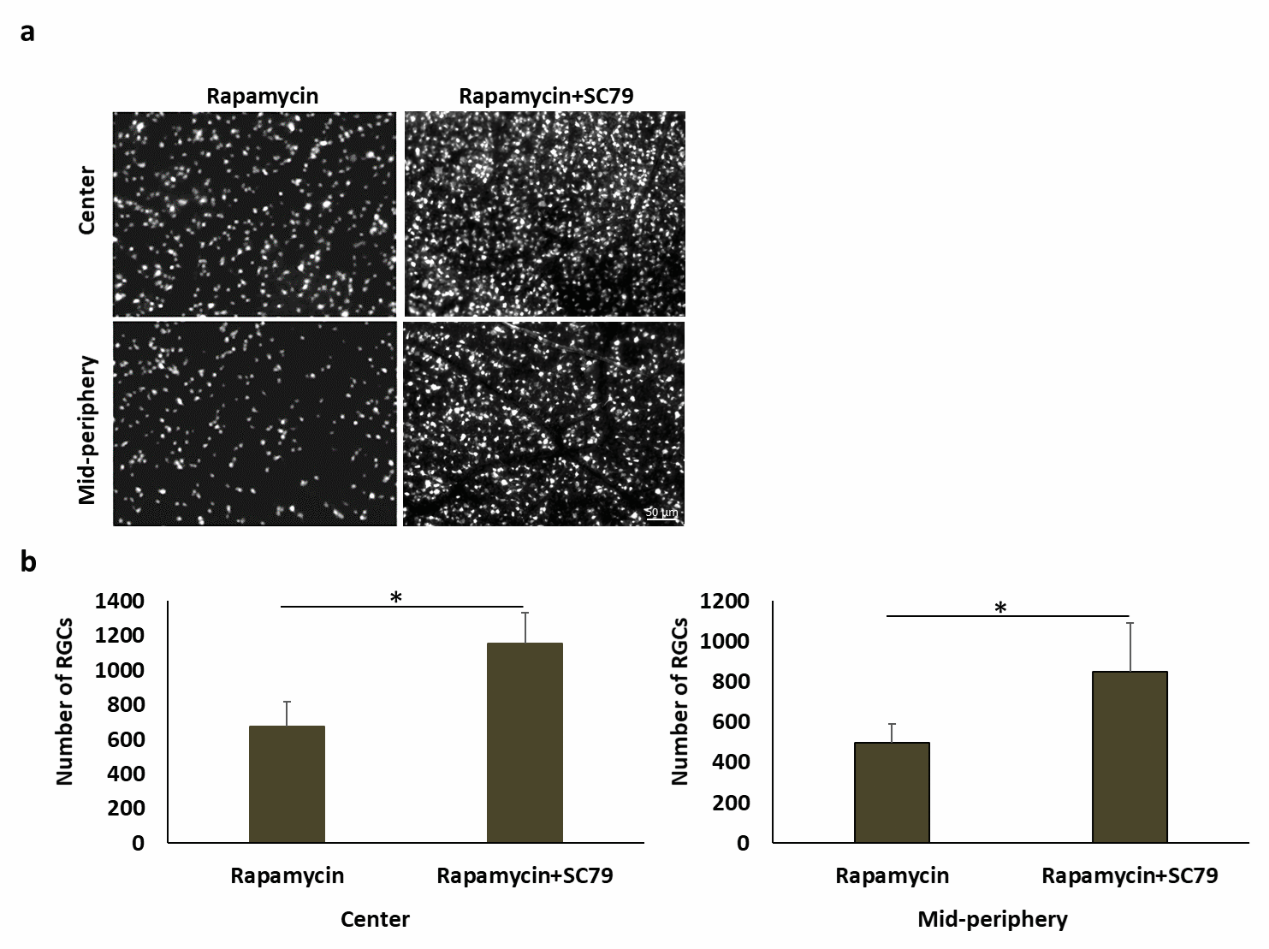


**Figure S2.** The better RGC preservation after combined treatment with rapamycin and SC79 compared to rapamycin treatment 2 weeks after ONC. (**a**) A representative of flat-mounted central and mid-peripheral retinas and the morphometry of RGCs in each group (n=6 per group) by a retrograde tracer (**b**) The RGC density of central retina and mid-peripheral retina in each group. Data are expressed as mean ± SD in each group (n=6). The number of RGCs in the central and mid-peripheral retinas was 1.72- and 1.70-fold higher in the combined treatment group than that in the rapamycin-treated group. *P<0.05.
